# Upregulated METTL3 promotes metastasis of colorectal Cancer via miR-1246/SPRED2/MAPK signaling pathway

**DOI:** 10.1186/s13046-019-1408-4

**Published:** 2019-09-06

**Authors:** Wen Peng, Jie Li, Ranran Chen, Qiou Gu, Peng Yang, Wenwei Qian, Dongjian Ji, Qingyuan Wang, Zhiyuan Zhang, Junwei Tang, Yueming Sun

**Affiliations:** 1 The First School of Clinical Medicine, Nanjing Medical University, Nanjing, People’s Republic of China; 2 Department of General Surgery, The First Affiliated Hospital of Nanjing Medical University, Nanjing, Jiangsu 210029 People’s Republic of China

**Keywords:** m6A modification, METTL3, miR-1246, Tumor invasion, CRC

## Abstract

**Background:**

m6A modification has been proved to play an important role in many biological processes. METTL3 as the main methyltransferase for methylation process has been found to be upregulated in many cancers, including CRC. Here, we investigate m6A modification and the underlying mechanism of METTL3 in the development of CRC.

**Methods:**

The expression of METTL3 was detected in large clinical patient samples. To evaluate the function of METTL3 in vitro and in vivo, colony formation, CCK-8, cell migration and invasion assays were performed. To find out the downstream target of METTL3, GEO dataset was re-mined. We analyzed expression and metastasis-related miRNA by Pearson correlation, and miR-1246 was selected. Here, to identify the downstream target of miR-1246, Targetscan and miRWalk were used. RIP and luciferase reporter assay further confirmed SPRED2 as the direct target of miR-1246.

**Results:**

We found that upregulated METTL3 is responsible for abnormal m6A modification in CRC and correlates positively with tumor metastasis. The gain- and loss-of-function indicates that METTL3 promotes cell migration and invasion in vitro and in vivo. Additionally, we confirmed that METTL3 can methylate pri-miR-1246, which further promotes the maturation of pri-miR-1246. By using bioinformatics tools, anti-oncogene SPRED2 was identified as the downstream target of miR-1246, wherein downregulated SPRED2 further reverses the inhibition of the MAPK pathway.

**Conclusions:**

The present study demonstrates that the METTL3/miR-1246/SPRED2 axis plays an important role in tumor metastasis and provides a new m6A modification pattern in CRC development.

**Electronic supplementary material:**

The online version of this article (10.1186/s13046-019-1408-4) contains supplementary material, which is available to authorized users.

## Background

Colorectal cancer (CRC) continues to be a critical health problem worldwide, with relatively high mortality and morbidity. The incidence of CRC is increasing in recent years and is ranked third for incidence but second in terms of mortality, with over 1.8 million new CRC cases and 881,000 deaths estimated to occur in 2018 [1]. Standard treatment for CRC is surgical resection followed by chemotherapy. However, the 5-year survival rate remains low and varies according to the specific stage [2, 3]. Distant metastasis is the main cause of sporadic death due to CRC, and the common metastasis sites are the liver, lung, etc. [4]. Although tremendous efforts have been made, the exact mechanisms of tumorigenesis and distant metastasis remain unclear. Therefore, more studies are urgently required to investigate the underlying molecular mechanism.

N6-methyladenosine (m6A) RNA methylation has emerged as an abundant and dynamically regulated modification and can be found in many coding or noncoding RNA at the consensus RRACU motif (R = G or A). In recent years, some discoveries have been made regarding the regulation and function of m6A, wherein m6A modification was found to play an important role in splicing process, stability, translation efficiency, and nuclear retention of mRNAs and noncoding RNAs [5, 6]. Three different types of protein complex determine the effects of m6A, including m6A readers, writers, and erasers. The addition of the methyl group is catalyzed by the writers, namely, m6A methyltransferase complex (MTC), including methyltransferase-like 3 (METTL3), methyltransferase-like 14 (METTL14), and Wilms’ tumor 1-associated protein (WTAP). Other proteins exert the function of demethylation to reverse methylation, such as Fat mass, obesity–associated protein (FTO) and ALKB homolog 5 (ALKBH5) [7, 8]. METTL3, the first reported m6A reader, was identified as the main methyltransferase for the methylation process and was further proven to affect the development of many cancers [9–12]. However, little researches are focused on the underlying mechanism of METTL3 in the development of colorectal cancer.

MicroRNA belongs to noncoding small RNA and contains 20–40 nucleotides. Most of them have important functions [13, 14]. Its biogenesis involves three steps. The first step is transcription, wherein the miRNA is processed in the nucleus to form pri-miRNA. Second, under the assistance of DGCR8 and Drosha, pri-miRNA transforms to pre-miRNA. Finally, pre-miRNA is exported to the cytoplasm for further processing. In the cytoplasm, pre-miRNA is cleaved by Dicer to become mature miRNA [15]. MiRNAs have been proved to regulate many cellular processes in animals by binding to 3’UTR, including RNA silencing and post-transcriptional regulation of gene expression [13, 16]. Especially in many diseases, diversity functions of miRNA have been reported; these include proliferation, apoptosis, migration, invasion, and differentiation [17, 18]. Because of its important role in eukaryotic cells, dysregulations of miRNA contribute to some activation of oncogenes or suppressors in cancer diseases [19–21].

In the present study, we elucidate the functional role of METTL3 in CRC. Our study demonstrates that upregulated METTL3 is associated with metastasis in vitro and in vivo through regulating the expression of miRNA-1246, which further suppress the expression of anti-oncogene SPRED2, thus providing a novel therapeutic target in CRC.

## Materials and methods

### Patient samples

Sixty patient samples were obtained from 2010 to 2011 at the department of general surgery, First Affiliated Hospital, Nanjing medical university, China. Written consents were approved by the patients enrolled in this study. The pathological information was obtained from Department of Pathology in First Affiliated Hospital of Nanjing Medical University, and the clinical stage was based on the 7th edition of the International Union Against Cancer (UICC) on Tumor-Node-Metastasis (TNM) staging system. Samples were all collected within 5 min after resected, then immediately transferred to a − 70 °C freezer for use. All experiments were performed in compliance with government policies and the Helsinki Declaration. The individuals were informed about the study and gave consent prior to the specimen collection.

### CRC cell lines and cell culture

CRC cell lines including LoVo, HCT116, CaCo2, DLD-1, HT-29 and NCM460 were purchased from the Cell Bank of Type Culture Collection of the Chinese Academy of Sciences (Shanghai, China) and were cultured under conditions specified by the manufacturers. HCT116 and HT-29 were cultured in McCoy’s 5A medium (Keygentec, Jiangsu, China) supplemented with 10% fetal bovine serum (FBS, Winsent, Quebec, Canada). LoVo was cultured in Ham’s F-12 K (Kaighn’s) Medium (Gibco, Carlsbad, CA, USA) supplemented with 10% FBS. DLD-1 was cultured in RPMI 1640 (Winsent, Quebec, Canada) supplemented with 10% FBS. CaCo-2 was cultured in Dulbecco’s modified Eagle’s medium (DMEM; Winsent, Quebec, Canada) supplemented with 20% FBS. NCM460 was cultured in DMEM supplemented with 10% FBS. All the medium contained 100 U/ml penicillin and 100 μg/ml streptomycin. And all the cells were incubated in a 5% CO2 humidified incubator at 37 °C.

### RNA extraction and quantitative real-time PCR (qRT-PCR)

Procedures were described previously [22]. Total RNA was extracted from tissues and cell lines using Invitrogen™ TRIzol reagent (Thermo Fisher Scientific, Inc., Waltham, MA, USA). Reverse transcription of mRNA was conducted using random primers through PrimeScript RT reagent kit (TaKara, Dalian, China). Bulge-loop™ miRNA qRT-PCR primer sets (one RT primer and a pair of qPCR primers for each set) specific for miR-1246 were designed by RiboBio (Guangzhou, China). The qRT-PCR experiment was performed using a SYBR Premix Ex Taq Kit (TaKaRa, Dalian, China) on an Applied Biosystems 7500 Sequence Detection System (Applied Biosystem). β-actin and U6 were used as internal control. The primers used were listed in Additional file 1: Table S1 The data was analyzed using the relative 2 ^-ΔΔCt^ method.

### Small interfering RNA transfection and lentiviral vectors

Small interfering RNA (siRNA) oligonucleotides targeting METTL3 and a negative were designed and synthesized by Genepharm (Shanghai China). Cells were transfected with siRNA using Lipofectamine3000 (Invitrogen, Thermo Fisher Scientific, Inc.) following the manufacturer’s instructions. The knockdown efficiency was evaluated by qRT-PCR and western blot after 48 h. Overexpression of METTL3 was conducted by using an expression plasmid (GV144, synthesized by GeneChem, Shanghai) and empty vector was used as the negative control. Short hairpin RNA (shRNA) targeting METTL3-specific regions was designed by GeneChem (Shanghai China) and cloned into the GV112 lentiviral vectors (GeneChem, Shanghai). The cells were transfected with the viruses in the presence of Polybrene. Forty-eight hours later, puromycin was added to medium for the selection of stable clones. The efficiency of overexpression was determined by qRT-PCR and western blots. The knockdown sequences were listed in Additional file 1: Table S2.

### RNA m6A quantification

Total RNA was extracted according to the manufacturer’s instructions. m6A RNA Methylation Quantification Kit (ab185912, Abcam) was used to determined RNA m6A level in total RNA. Firstly, RNAs were coated to the assay wells for 90 min at 37 °C. And then adding capture antibody, detection antibody and enhancer solution separately. Finally, adding color developing solution Signal Detection and measuring absorbance. The m6A levels were quantified calorimetrically by reading the absorbance of each well at a wavelength of 450 nm, and calculations were performed based on the standard curve.

### m6A dot blot assays

Briefly, poly(A) RNA was isolated from total RNA using the Dynabeads® mRNA Purification Kit (61,006, Invitrogen) following the manufacturer’s instructions and then a serial dilution of denatured RNA was spotted onto a the Hybond-N+ membrane (GE Healthcare). The membranes were then UV crosslinked, blocked, incubated with m6A antibody (Abcam) and horseradish peroxidase conjugate anti-rabbit immunoglobulin G, and then the membrane was incubated with 3 ml of western blotting substrate. Finally, the signals from the dot blot was visualized by an ECL Western Blotting Detection Kit (Thermo).

### Immunohistochemistry

IHC was conducted as previously described [23]. All tumor samples were embedded in paraffin and sectioned into 4-mm slices. The sections were incubated with primary antibody against METTL3 (Abcam). Finally, the photograph of sections was detected using a digital microscope camera.

### Western blot

Proteins were collected according the manufactory’s protocol (KeyGEN BioTech). Protein concentrations were measured using the BCA Protein Assay Kit (Beyotime Biotechnology). The antibody information was listed in Additional file 2: Table S5.

### Transwell and invasion assays

Transwell and invasion assays were conducted using Millicell cell culture inserts (24-well insert, 8-μm pore size) according to the manufacturer’s instructions. For migration assay, 4 × 10^4 cells (per well) in 200 μL serum-free medium were loaded into the bottom of the inserts, then the lower chambers were filled with 500ul medium supplemented with 10% FBS. For invasion assay, Matrigel (BD Biosciences, Franklin Lakes, NJ, USA) was coated into inserts, and then 8 × 10^4 cells (per well) in serum-free medium were loaded. Medium containing 10% FBS was added to the lower chamber. After 24 h (48 h based on different cell lines) of incubation, cells on the underside of the membrane were fixed and stained with 0.5% crystal violet solution. Five random fields were counted in each well under a microscope.

### Scratch wound healing assay

Briefly, 5 × 10^5 cells (per well) were seeded into 6-well culture plate. After incubation for 48 h, scratches were performed into the middle slides using a sterile 200ul pipette tip. Growing cells for another 48 h, photographs were taken to estimate closure of the gap. The gap distance was quantitatively evaluated using ImageJ.

### Dual-luciferase reporter assay

Reporter assay was conducted as previously described [24]. To test the binding specificity, the wide and mutant SPRED2 3′-UTR were inserted into the reporter plasmid. Cells seeded in 24-well plate were co-transfected with plasmids and miR-1246 mimics or negative control by using lipofectamine 3000 (Invitrogen). After incubation for 48 h, the firefly and Renilla luciferase activities were measured using the Dual Luciferase Reporter Assay System (Promega, WI, USA). The luciferase activities were normalized to Renilla fluorescence.

### Mutagenesis assay

Point mutations were achieved using the QuikChange Lightning Multi Site-Directed Mutagenesis Kit (Agilent Technologies) following the manufacture’s instruction. Mutagenic primer was strictly designing to fulfill requirement in the web-based quikchange primer design program (www.agilent.com/genomics/qcpd) (Additional file 1: Table S3). The plasmid DNA template was isolated from dam^+^ *E. coli* strain. We cycled the mutant strand synthesis reactions using the standard parameters. And then adding 1ul Dpn1 restriction enzyme to digest the amplification products. We transferred proper amount of Dpn1-treated DNA to the ultracompetent cells for transformation reaction. Finally, the products of transformation reaction were plating on agar plates containing antibody for plasmid vector. The point mutation results were confirmed by sequencing.

### RNA immunoprecipitation (RIP) assay

RIP assay was adapted from a previous publication [12]. A Magna RIP RNA-Binding Protein Immunoprecipitation Kit (Millipore, USA) was used. Briefly, cells were collected and lysed by the RIP lysis buffer. Then the corresponding antibodies were added into the cleared lysates and incubated in the magnetic beads’ suspension with rotating overnight at 4 °C. Precipitate was digested with Proteinase K buffer, and then co-immunoprecipitated RNA was isolated for qRT-PCR and miRNA RT-PCR analysis, respectively.

### m6A RNA immunoprecipitation (MeRIP) assay

MeRIP was performed using the Magna MeRIP m6A Kit (Millipore, USA) according to the manufacturer’s instructions. Briefly, 3μg of anti-m6A antibody (Abcam) was conjugated to protein A/G magnetic beads overnight at 4 °C. And then the antibody conjugated beads were incubated with the antibody in IP buffer with RNase inhibitor and protease inhibitor. The interacting RNAs were isolated and detected by qRT-PCR.

### In vivo metastasis assays

Male BALB/c nude mice aged 5 weeks were purchased from the animal center of Nanjing Medical University. All animal experiments were performed under the experimental animal use guidelines of the National Institutes of Health. To evaluate the in vivo metastasis abilities, the method described before was performed [25]. Briefly, the spleen in the upper left lateral abdomen of the anesthetized mice were exposed, 10^6^ cells suspended in 20 μL phosphate-buffered saline (PBS) were injected into the distal tip of the spleen. After injection, replacing the spleen, and closing the incision. The animals were sacrificed after six weeks, and the livers were dissected out and embedded in paraffin.

### Statistical analysis

Each experiment was performed at least 3 times. Statistical analyses were performed using the GraphPad Prism5 software. Student’s t-test (two tailed), and ANOVA were used to detect differences between two groups or more than two groups. Chi-square test was used to estimate the correlation between METTL3 expression and clinicopathologic features. Pearson correlation was used to analysis the correlation between expression of METTL3 – miR-1246, and miR-1246 – SPRED2. Results of Western blot and wound healing assay were quantified by ImageJ software (National Institutes of Health). All data were shown as mean ± standard deviation (SD).

## Results

### Increased m6A level in CRC

**Fig. 1:**
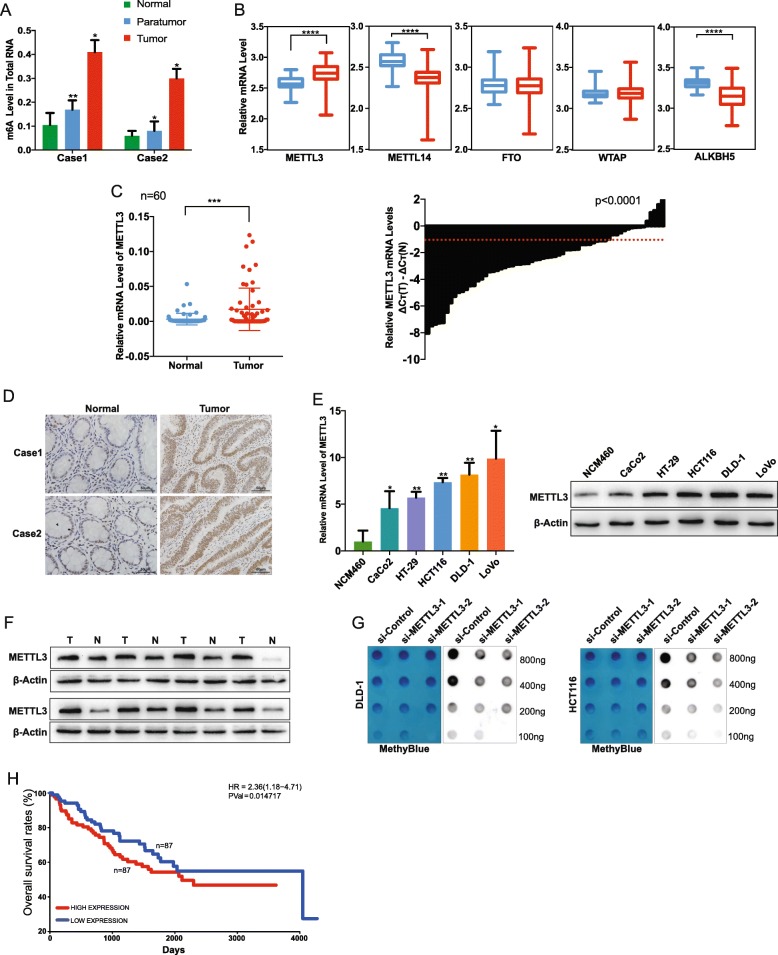
Upregulated expression of mettl3 was responsible for abnormal m6a modification in CRC. (**a**) m6A modification was detected using m6a quantification in two samples. (**b**) Catalytic proteins involved in m6a modification were assessed in CRC (TCGA data, blue box for normal tissues, *n* = 379; red box for tumor tissues, *n* = 51). (**c**) Relative increased level of METTL3 was confirmed in the CRC tumor and adjacent normal tissues by qRT-PCR (*n* = 60). (**d**) Representative image of immunohistochemical staining by METTL3 antibody in two patient tissues. Original magnification 100X; scale bar: 50 μm. (**e**) qRT-PCR showed the expression level of METTL3 in CACO2, HT-29, HCT116, DLD-1, LOVO and NCM460; western blot showed the protein level in cell lines above. (**f**) The expression of METTL3 in eight pairs CRC tissues (T) and adjacent normal tissues (N) were detected by western blot. (**g**) Poly(A) + RNA isolated from METTL3-knockdown cells were used in dot blot assay with m6A antibody. Methylene blue staining served as a loading control. (**h**) Kaplan-Meier survival curves of OS based on METTL3 mRNA expression in 174 CRC patients. All patients were divided into two groups based on the median level of METTL3. The log-rank test was used to calculate the significant level (GEO data). Data are presented as means ± standard deviation (*P < 0.05, **P < 0.01, ***P < 0.001, ****P < 0.0001)

To investigate the role of m6A modification in CRC in vitro, we detected m6A level in two human CRC samples by using the colorimetric m6A quantification assay. Results showed that the m6A levels in both tumor tissues and their corresponding adjacent tissues were significantly increased as compared to that in normal tissues (Fig. 1a). Furthermore, the m6A level in both tumor tissues were elevated than that in their corresponding adjacent tissues (Fig. 1a). These findings suggested that m6A levels were increased in human CRC.

### METTL3 was upregulated in human CRC tissues and cell lines

Because of the pivotal role of m6A methyltransferases and demethylases in m6A modification, we hypothesized that the increased m6A level in CRC was caused by dysfunction of these m6A modification enzymes. Many key catalytic proteins are involved in the dynamic m6A modification, including METTL3, METTL14, WTAP, FTO, ALKBH5, and KIAA1429. To validate our assumption, data downloaded from TCGA were re-analyzed (Fig. 1b), and it was found that only the expression of METTL3 was significantly elevated in tumor tissues as compared to that in normal tissues; in contrast, the expression of METTL14 showed a significant decrease, while the expression of other proteins was not significantly changed. To further assess the results, we obtained 60 paired tumor tissues and adjacent normal tissues of CRC, and qRT-PCR was used to determine the expression pattern of METTL3. The results indicated that the expression of METTL3 was significantly higher in CRC tissues than in adjacent normal tissues (Fig. 1c, left panel). METTL3 was upregulated in (41/60) CRC samples; this finding is in line with the TCGA data (Fig. 1c, right panel). Furthermore, IHC results showed that the expression of METTL3 in tumor tissues was dramatically elevated compared to that in normal tissues (Fig. 1d). We assessed the expression of METTL3 in five CRC cell lines (DLD-1, HT-29, HCT116, CaCo2, and LoVo) and normal colorectal mucosa epithelial cells (NCM460) at both mRNA and protein levels (Fig. 1e). The expression of METTL3 was higher in the five CRC cell lines than in NCM460. We then analyzed the METTL3 protein level in eight samples by western blot analysis. As expected, the METTL3 level was higher in tumor tissues than in the adjacent normal tissues (Fig. 1f).

**Fig. 2:**
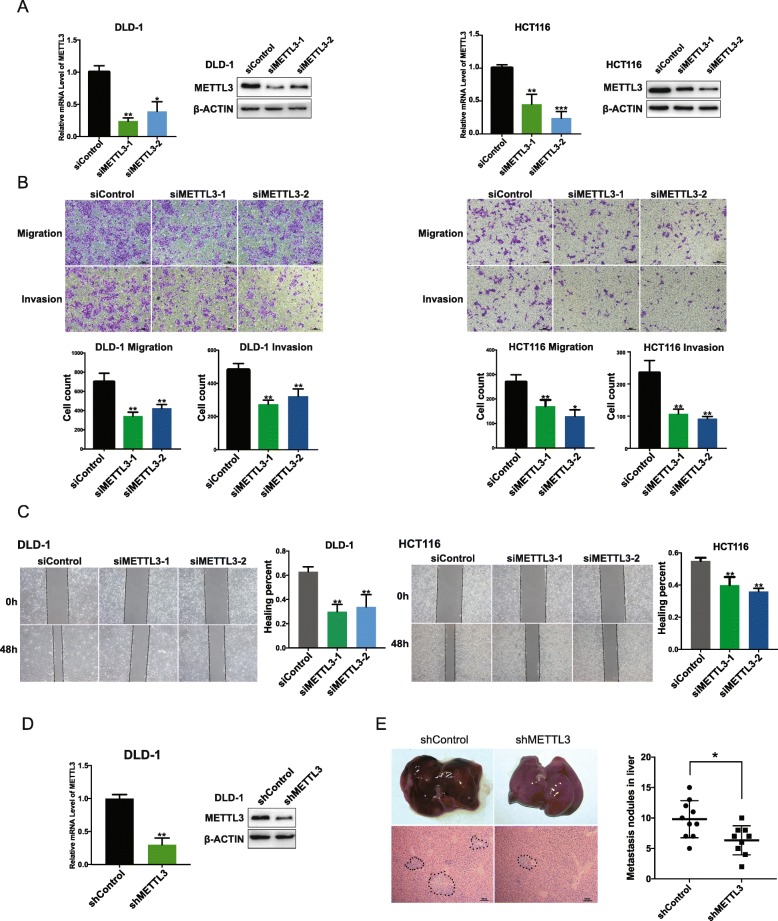
Knocking down the expression of METTL3 impaired the migration and invasion ability of CRC cell lines in vitro and in vivo. (**a**) Left and right panel showed the expression level of METTL3 in DLD-1 and HCT116 cell lines transfected with siRNA determined by qRT-PCR and western blot. (**b**) Effect of knocking down the expression of METTL3 on transwell assay; Representative graphs are shown. Original magnification 200X; scale bar: 50 μm. (**c**) Effect of knocking down METTL3 level on scratching healing assay; The scratch was measured 48 h later; Representative graphs are shown. Original magnification, 40X; scale bar: 100 μm.(**d**) METTL3 level in DLD-1 cell line transfected with lentiviral shRNA. (**e**) Hepatic metastasis foci in metastasis model graphs were shown. Right panel showed the metastasis foci formed in the liver were compared between shControl and shMETTL3. Black dot indicated the metastatic foci. Data are presented as means ± standard deviation (*P < 0.05, **P < 0.01)

To investigate whether abnormal METTL3 expression was responsible for the increased m6A level in CRC cell lines, we transfected DLD-1 and HCT116 cell lines with two different siRNAs (Fig. 2a). As expected, decreased METTL3 could result in lower m6A level in CRC cell lines (Fig. 1g). To further characterize METTL3 expression and clinical features, the expression level was classified into two groups on the basis of their median value (High: *n* = 30, Low: n = 30). High expression of METTL3 was correlated with lymph node invasion, and distant metastasis (Table 1). Furthermore, from a metastasis gene expression profile downloaded from GEO (GSE17536), patients with high expression of METTL3 showed lower overall survival (Fig. 1h, right). On the basis of these findings, we concluded that METTL3 is upregulated in human CRC and involved in abnormal m6A modification of CRC and could be a prognostic factor in CRC.

**Table 1:** Relevance of Analysis of METTL3 Expression in CRC Patients

METTL3 Level					
Characteristics	*n*	Low	High	*X* ^2^	*P*
Age (years)					
< 60	19	10	09	0.075	0.786
> 60	41	20	21		
Gender					
Male	33	14	19	1.674	0.201
Female	27	16	11		
Lymph node metastasis					
Negative	28	19	09	7.286	0.009**
Positive	32	11	21		
Distant metastasis					
Negative	40	24	16	5.043	0.029*
Positive	20	06	14		
Tumor stage					
Stage 1,2	24	15	09	2.552	0.118
Stage 3,4	36	15	21		
TNM staging system					
T1 + T2	29	18	11	3.344	0.073
T3 + T4	31	12	19		
Tumor size (cm)					
> 5	26	12	14	0.264	0.610
< 5	34	18	16		

NOTE: A chi-square test was used for comparing groups between low and high METTL3 expression. *P < 0.05, **P < 0.01

### METTL3 downregulation impaired the metastatic capacity of CRC

To investigate the pivotal role of METTL3 in CRC, two cell lines were selected, including DLD-1 and HCT116, for further research, on the basis of their expression pattern. We then silenced METTL3 in DLD-1 and HCT116 cells with two distinct siRNA (siMETTL3–1 and siMETTL3–2). The knockdown efficiency was assessed by qRT-PCR and western blot, and the results showed that METTL3 expression was knocked down effectively by siRNA in both DLD-1 and HCT116 cell lines (Fig. 2a). Proliferation ability was measured by colony formation, CCK8, and EdU assay. However, no significant results were found in these assays (data not shown). Transwell assay and scratch wound healing assay were then performed to detect whether METTL3 depletion could affect migration and invasion ability. Transwell assay results showed that METTL3 depletion significantly impaired the migration and invasion of CRC cells as compared to that of the control group (Fig. 2b); scratch wound healing assay also showed the same results (Fig. 2c). Besides that, a metastatic cell line (LoVo) was brought to the experiments. The same two siRNAs were used to knockdown the expression of METTL3 in LoVo cell line, and results were showed in Additional file 4: Fig. S3 Consistent with the results of knocking down of METTL3 in HCT116 and DLD-1 cell lines, the knock-down of METTL3 in LoVo cells presented the suppression of cell migration and invasion.

To determine the in vivo role of METTL3 in CRC, stable METTL3-knockdown cell line was constructed using lentivirus (shMETTL3), and the knockdown efficiency was confirmed through qRT-PCR and western blot (Fig. 2d). To investigate the inhibition of metastasis effect in vivo, cells were injected into the distal tip of the spleen as described before. Six weeks later, all mice were sacrificed, and their livers were resected and embedded in paraffin. We then evaluated the metastases nodules in the liver. All the mice injected with shControl or shMETTL3 cell line showed liver metastasis (9 of 9 in shMETTL3 group, and 10 of 10 in shControl group). Both the shControl and shMETTL3 cells grew locally in the spleen at the site of injection to form a primary tumor. Moreover, we found that more metastatic nodules were formed in shControl group mice compared with shMETTL3 group (Fig. 2e).

Taken together, all the data showed that METTL3 downregulation impaired the metastatic capacity of CRC in vitro and in vivo.

### METTL3 upregulation enhanced the metastatic capacity of CRC

**Fig. 3:**
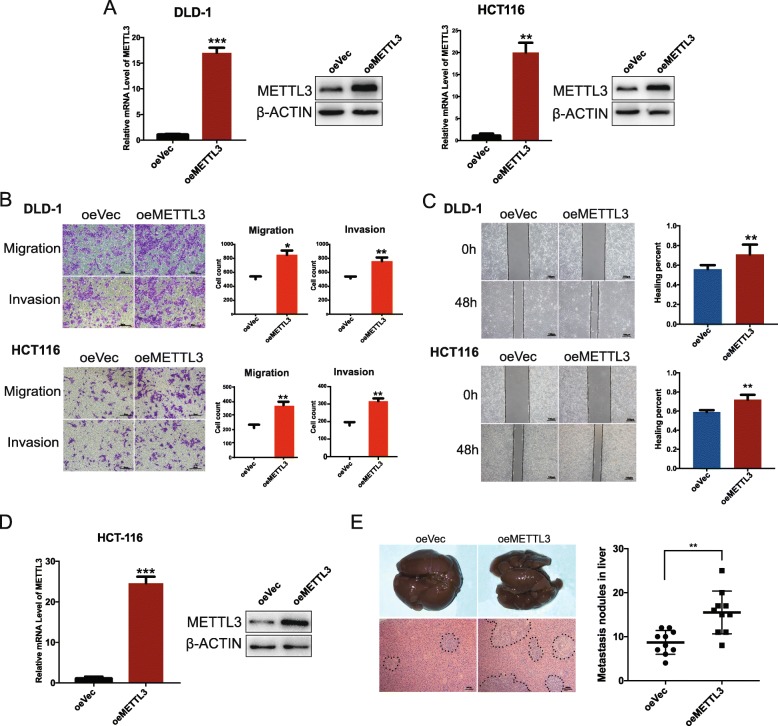
Overexpression of METTL3 promoted the migration and invasion ability of CRC cell lines in intro and in vivo. (**a**) Overexpression of METTL3 in DLD-1 and HCT116 cell lines. (**b**) transwell migration and invasion assays in METTL3-overexpressing cell lines. Original magnification 200X; scale bar: 50 μm. (**c**) Scratch wound healing assay in METTL3-overexpressing cell lines. Original magnification, 40X; scale bar: 100 μm. (**d**) Stable overexpression of METTL3 in HCT-116 cell line, the METTL3 level was shown (**e**) Hepatic metastasis foci in metastasis model graphs were shown. Right panel showed the metastasis foci formed in the liver were compared between oeVec and oeMETTL3 group. Black dot indicated the metastatic foci. Data are presented as means ± standard deviation (*P < 0.05, **P < 0.01)

Next, we constructed METTL3-overexpressing plasmid (oeMETTL3). The upregulation efficiency was confirmed at both mRNA and protein levels (Fig. 3a). METTL3 overexpression significantly promoted the migration and invasion capacity of DLD-1 and HCT116 cell lines (Fig. 3b). The wound healing assay showed similar results (Fig. 3c), thus demonstrating that the migration capacity of both cell lines enhanced by approximately 30 and 39%, respectively. We then constructed the stable METTL3 overexpressed cell line (HCT116). The stable overexpression efficiency was assessed by qRT-PCR and western blot (Fig. 3d). For in vivo assay, procedures were performed as protocol above, we then evaluated the metastases nodules in the liver. Results showed that all mice developed metastases nodules (10 of 10 in oeMETTL3 group, and 10 of 10 in oeVec group), further analysis indicated that more nodules were formed in oeMETTL3 group compared with oeVec group (Fig. 3e).

These results showed that METTL3 upregulation enhances the metastatic capacity of CRC.

### METTL3 enhanced the metastatic potential by promoting the expression of miR-1246

Previously, Alarcon et al. proved that m6A mark was a key post-transcriptional modification that promoted the initiation of miRNA biogenesis. METTL3 could methylate pri-miRNAs at certain sites that were enriched within pri-miRNA, thus marking them for recognition and processing by DGCR8 [26]. This suggested that altered METTL3 expression in various human malignancies may contribute to the up- or downregulation of many miRNAs. Because miRNAs have been proved to be involved in tumorigeneses, we hypothesized that METTL3 may target metastasis-related miRNA and further influence the migration and invasion ability of CRC cells in a pri-miRNA-processing way.

**Fig. 4:**
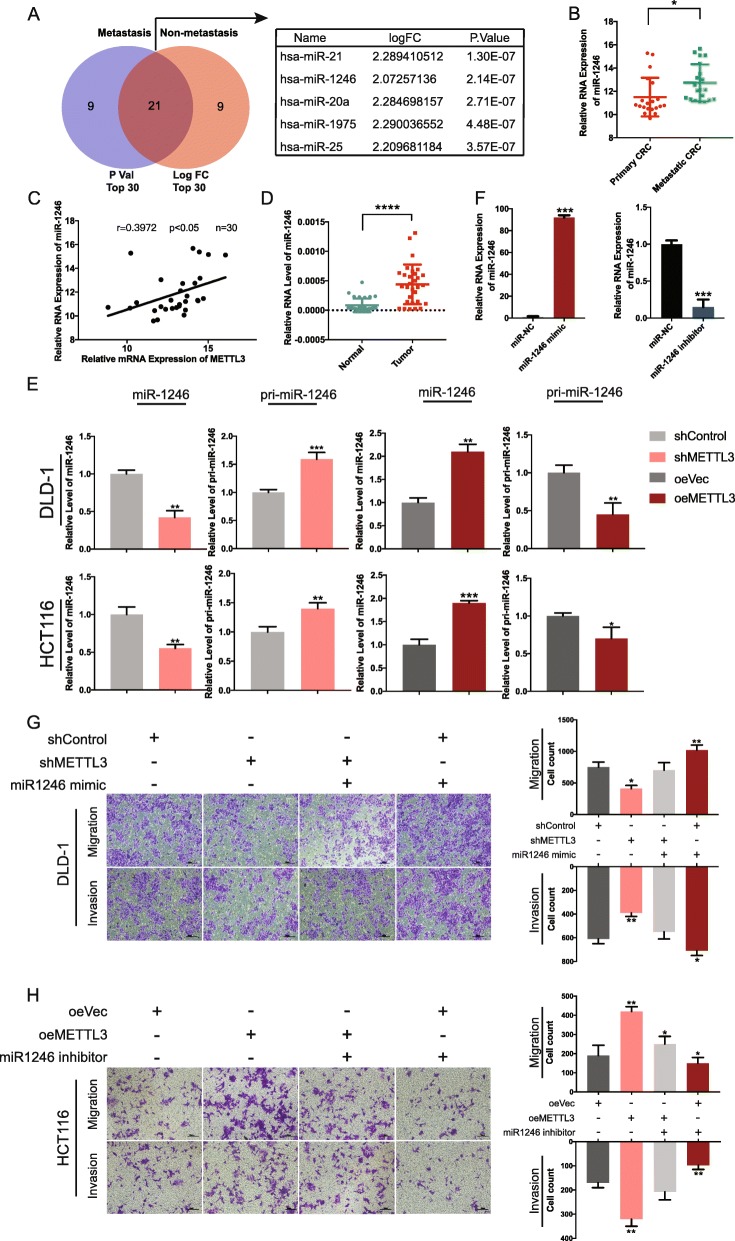
Upregulated METTL3 enhanced the metastatic ability through modulating the maturation of pri-miR-1246. (**a**) Venn diagram showed the overlapping of metastasis-related miRNA from the top 30 ranked by P value and Log FC (GEO data, *P* < 0.05, Log FC > 1) (left panel); The top five miRNA were listed in the table (right panel). (**b**) qRT-PCR was used to measure the expression level of miR-1246 in 20 cases of primary CRC tissues with metastasis and 20 cases of primary CRC tissues without metastasis. (**c**) Scatter plot were showed between miR-1246 and METTL3 in CRC (r = 0.3972, *p < 0.05, *n* = 30). (**d**) Relative level of miR-1246 between tumor and adjacent normal tissues (*n* = 30). (**e**) qRT-PCR quantification of miR-1246 and pri-miR-1246 level that were affected by METTL3 depletion and overexpression. (**f**) Knockdown and overexpression level of miR-1246 were assessed by qRT-PCR. (**g-h**) The transwell migration assay in control and METTL3-depletion DLD-1 cell line or METTL3-overexpressing HCT116 cell line in the presence of miR-1246 mimics or miR-1246 inhibitors. Original magnification 200X; scale bar: 50 μm. Data are presented as means ± standard deviation (*P < 0.05, **P < 0.01, ***P < 0.001, ****P < 0.0001)

To validate our assumption, we first downloaded microarray data of CRC from the Gene Expression Omnibus database of the National Center for Biotechnology Information (GSE120300). Metastasis-related miRNAs were compared among the metastatic and non-metastatic tumor tissues, and the Venn diagram showed 21 overlapping miRNAs ranked by logFC and *P* values (Fig. 4a, left panel). We selected top-five miRNAs according to their fold-change and P values (Fig. 4a, right panel). qRT-PCR was used to assess these metastasis-related miRNAs, and results demonstrated that candidate miR-1246 showed significant differences in our samples between metastatic and non-metastatic patients (Fig. 4b). Further, levels of miR-1246 and levels of METTL3 mRNA in CRC tissues exhibited a significant positive correlation calculated by Pearson correlation test (Fig. 4c). Besides, miR-1246 expression was higher in tumor tissues than in adjacent normal tissues (Fig. 4d). And Kaplan-Meier Plotter analysis (http://kmplot.com/analysis/) indicated that higher miR-1246 expression correlated with worse OS (Additional file 3: Fig. S1).

To determine whether METTL3 could regulate the maturation of miR-1246, we knocked down and overexpressed METTL3 respectively. Consistent with our assumption, when METTL3 was knocked down, the expression of mature miR-1246 was decreased, while the level of pri-miR-1246 was significantly upregulated. In contrary, pri-miR-1246 was downregulated, while mature miR-1246 increased when METTL3 was inhibited (Fig. 4e). These findings revealed that METTL3 promoted the transition from pri-miR-1246 to mature miR-1246. We then investigated whether miR-1246 was a downstream target of METTL3. We treated METTL3 knocked down and overexpressed cell lines with miR-1246 mimic or inhibitor, and the knockdown and overexpression levels were determined by qRT-PCT (Fig. 4f). We found that downregulated capacity of migration and invasion could be reversed in knocked down DLD-1 cell line using miR-1246 mimics (Fig. 4g). Further, miR-1246 inhibitor could also reverse the upregulation of migration and invasion in the overexpressed HCT116 cell line (Fig. 4h).

**Fig. 5:**
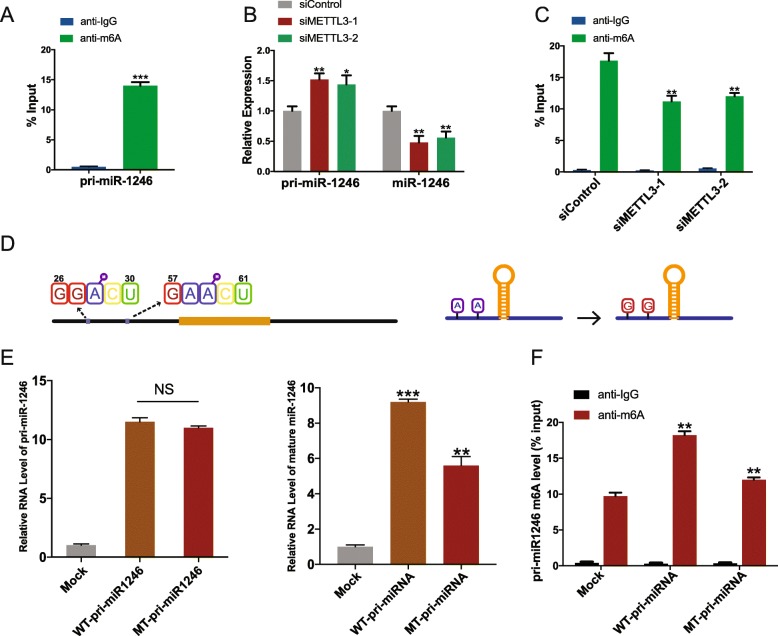
METTL3 regulated the maturation of pri-miR-1246 by changing the m6A modification status of pri-miR-1246. (**a**) m6A modification was enriched in pri-miR-1246; The percentage of the input is shown. (**b**) The expression level of pri-miR-1246 and miR-1246 when METTL3 was knocked down in DLD-1 cell line. (**c**) m6A modification level in the presence of METTL3 depletion. (**d**) Right panel showed RRACU m6A motifs sites in the upstream of pri-miR-1246; Left panel showed the motif mutation. (**e**) Relative level of pri-miR-1246 upon transfected with wide-type pri-miR-1246 or mutant plasmids (left panel) in HEK293T cell line; Relative level of mature miR-1246 upon transfected with wide-type or mutant pri-miR-1246 plasmids (right panel) in HEK293T cell line. (**f**) Changes in m6A modification level of pri-miR-1246 between wide-type and mutant-type plasmids. Data are presented as means ± standard deviation (**P < 0.01, ***P < 0.001)

We then investigated m6A modification status of pri-miR-1246 by performing MeRIP assay. Results showed that m6A modification is enriched with pri-miR-1246 sequence (Fig. 5a). When METTL3 was knocked down, consistent with the previous study results, the expression level of pri-miR-1246 was upregulated while miR-1246 level was decreased (Fig. 5b). Nevertheless, the m6A modification of pri-miR-1246 was significantly decreased (Fig. 5c). For further research, we obtained the full sequence of pri-miRNA-1246 and pre-miRNA-1246 from UCSC (Additional file 1: Table S4). The pri-miRNA-1246 contained 273 nucleotides. We analyzed these 273 nucleotides and found 2 potential RRACU m6A motifs, which had been proved to be the m6A deposition site in the upstream of miR-1246 (Fig. 5d, left panel). To determine whether these sites participated in the m6A modification of pri-miRNA-1246, we constructed expression vectors that contained m6A motif mutants and wild types. In the mutant-type vector, adenine in the m6A motif was replaced with guanine (Fig. 5d, right panel). We then transfected HEK293T cell line with equal amounts of wild-type and mutant-type pri-miRNA-1246 vectors. Transcript level of pri-miR-1246 was observed to be unchanged between wild-type and mutant-type cells (Fig. 5e, left panel), but significant differences were observed in the expression level of mature miRNA-1246 between these groups (Fig. 5E, right panel). We also found decreased m6A level in mutant type of pri-miRNA-1246 as compared to that in the wild type (Fig. 5f). These findings confirmed that the sites we predicted were responsible for m6A modification of pri-miR1246.

### SPRED2 served as a downstream target of miR1246 in CRC

**Fig. 6:**
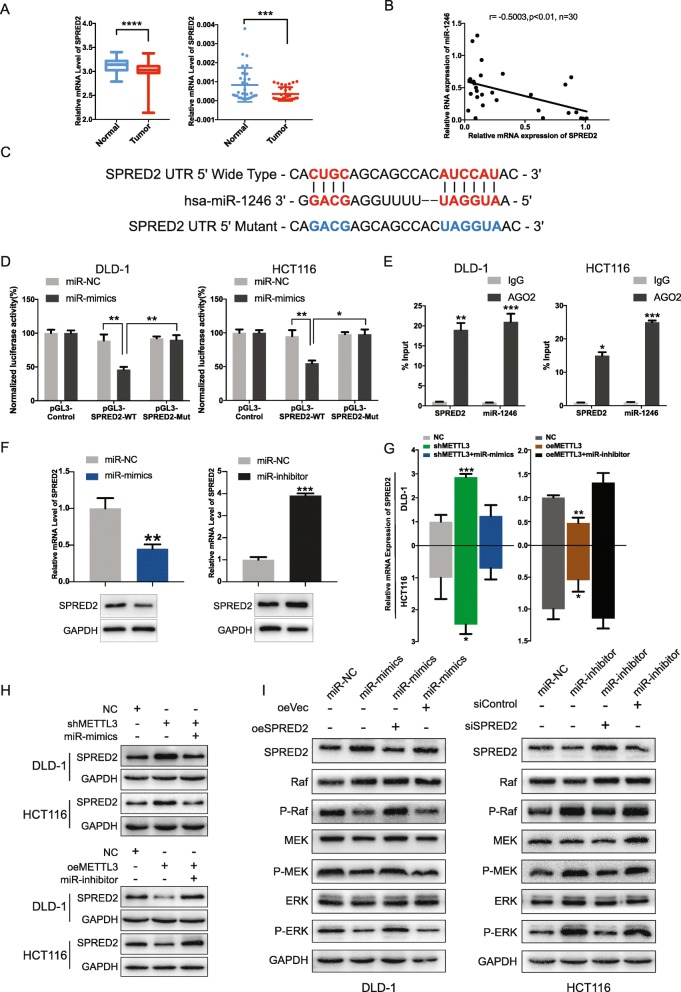
miR-1246 suppressed the expression of SPRED2 by binding to its 3’UTR. (**a**) Relative level of SPRED2 between normal and tumor tissues (TCGA data, 50 normal cases and 378 tumor cases) (left panel); The expression of SPRED2 was assessed by qRT-PCR between tumor and adjacent normal tissues in our patient samples (right panel) (*n* = 30). (**b**) The expression of SPRED2 and miR-1246 were analyzed in 30 cases of clinical CRC tissues (Pearson’s correlation coefficient, r = 0.5003, *P < 0.01, n = 30). (**c**) The binding sites of miR-1246 in the 3’UTR of SPRED2 mRNA and the wide type or mutant type of binding site were shown. (**d**) The effect of miR-mimics on the pGL3-SPRED2-WT and pGL3-SPRED2-Mut reporters in DLD-1 and HCT116 cell lines was measured by dual luciferase reporter gene. (**e**) RIP assay confirmed the binding between SPRED2 and miR-1246 in DLD-1 and HCT116 cell lines. (**f**) Relative level of SPRED2 when treated with miR-1246 mimics or miR-1246 inhibitors. (**g**, **h**) Relative level of SPRED2 was detected by qRT-PCR and western blot treated with miR-mimics or miR-inhibitors in the presence of stable knocked-down and overexpressed METTL3 cell lines. (**i**) The phosphorylation status of RAF/MEK/ERK pathway when co-transfected with oeSPRED2 plasmids and miR-1246 mimics or siSPRED2 and miR-1246 inhibitors. Data are presented as means ± standard deviation (*P < 0.05, **P < 0.01, ***P < 0.001, ****P < 0.0001)

The main mechanism by which miRNAs exert their function is through regulation of gene expression by using RNA-induced silencing complex. Here, to identify the downstream target of miR-1246, Targetscan and miRWalk were used. SPRED2 was chosen for further research for its anti-oncogene role in tumorigenesis, and low expression of SPRED2 was noted in tumor tissue than in the adjacent normal tissues in both TCGA and our patient tissues (Fig. 6a). Correlation analysis also showed a negative correlation between the SPRED2 and miR-1246 expression levels in 30 CRC tissues (Fig. 6b). To further probe the direct relationship between miR-1246 and SPRED2, we constructed dual luciferase reporters containing the 3’UTR of SPRED2 and the mutant type 3’UTR of SPRED2 (Fig. 6c). The results showed that miR-1246 mimics significantly reduced the luciferase activities of SPRED2 wild-type reporters compared with the mutant-type reporters (Fig. 6d). We then used the key protein Ago2 of RNA-induced silencing complex to immunoprecipitate miR-1246 and SPRED2. As expected, miR-1246 and SPRED2 were enriched in Ago2-binding RNAs as compared to the negative control (Fig. 6e). In addition, we knocked down and overexpressed miR-1246. The expression of SPRED2 was increased significantly when miR-1246 was knocked down and decreased when miR-1246 was overexpressed at both mRNA and protein levels (Fig. 6f). The above results demonstrated that miR-1246 could directly bind to SPRED2 and negatively regulated the expression of SPRED2.

To validate the existence of the METTL3/miR-1246/SPRED2 axis, we transfected DLD-1 and HCT116 with miR-1246 mimics and miR-1246 inhibitors in the presence of stable knocked down and overexpressed METTL3. The results showed that increased SPRED2 level in the shMETTL3 group could be reversed in cells co-transfected with shMETTL3 and miR mimics. In contrast, the decreased expression of SPRED2 in the oeMETTL3 group could be reversed in cells co-transfected with oeMETTL3 and miR inhibitors at both mRNA and protein levels (Fig. 6g, h). SPRED2 was reported to have an antioncogene role in tumors, wherein it regulates growth factor-induced activation of the mitogen-activated protein kinase (MAPK) cascade. We then detected the phosphorylation status in the MAPK signaling pathway. Western blotting showed that overexpression of SPRED2 and inhibition of miR-1246 expression could increase RAF/MEK/ERK phosphorylation (Fig. 6i), while miR-1246 mimics and siSPRED2 hindered the activation of RAF/MEK/ERK (Fig. 6i). These findings proved that METTL3 regulated the metastasis capacity of CRC by the METTL3/miR-1246/SPRED2 axis through activation of the RAF/MEK/ERK pathway.

## Discussion

In recent years, m6A modification has become another epigenetic hot point. First discovered in 1970s, m6A research was revived in 2000s, due to the limitation of research methods [27]. Dominissini et al. (2012) and Meyer et al. (2012) introducing a brand-new sequencing method to map m6A through the transcriptome [5, 28]. Since then, m6A modification was intensely investigated.

m6A modification is a dynamic process, from demethylated form to methylated form and vice versa; each process is regulated precisely by different proteins. m6A modification participates in many biological processes in mammals, such as RNA splicing, protein translation, and stem cell renewal [10, 29, 30]. Many recent studies have partly revealed the underlying mechanisms of m6A modification in cancers. Lin and Choe et al. reported that m6A methyltransferase METTL3 interacts with translation initiation machinery to promote translation [31], which further influenced the growth, survival, and invasion of cancer cells. Cai and Wang et al. concluded that increased METTL3 promoted the progression of breast cancer by inhibiting miRNA let-7 g [12]. Visvanathan and Patil et al. revealed the essential role of METTL3 as a potential molecular target in glioblastoma (GBM) therapy [9].

However, little researches were focus on the underlying mechanism of METTL3 in the development of CRC. Recently, the role of METTL3 in CRC has recently been partly investigated by Deng R and Li T respectively. Deng R etc. reported the tumor-suppressive role in CRC cell proliferation, migration, and invasion through p38/ERK pathways [32]. In contrary to Deng’s results, Li T etc. revealed that METTL3, acting as an oncogene, maintained SOX2 expression through an m6A-IGF2BP2-dependent mechanism in CRC cells [33]. Here, we identified METTL3 as a pivotal regulator in CRC, which promoted the development of tumor metastasis. Consistent with previous reports of METTL3 as an oncogene in CRC [33], we found that METTL3 was upregulated in CRC, and its abnormal expression could result in aberrant m6A modification. Furthermore, increased METTL3 was significantly associated with prognosis. A functional study revealed the essential role of METTL3 in promoting CRC migration and invasion in vitro and in vivo. More importantly, we found that METTL3 regulated tumor metastasis by processing the maturation of pri-miR1246 in a DGCR8-dependent manner, which was previously reported by Alarco’n and Lee et al. [26]. In addition, we identified SPRED2 as the downstream target of miR-1246. As an anti-oncogene, SPRED2 interacted with the Raf/MEK/ERK pathway to prevent cancer cell migration and invasion [34–36]. Here, we found METTL3-mediated upregulation of miR-1246 negatively regulated the expression of SPRED2, which further inactivated the Raf/MEK/ERK pathway.

Further, the same results could be seen in other cancer cell line (e.g. SGC-7901, a gastric cancer cell line), knocking down of METTL3 also resulted in the suppression of cell migration or invasion abilities, besides that, the suppression of cell proliferation could also be observed (Additional file 4: Fig. S4).

Other mechanisms by which METTL3 promotes tumor metastasis need to be urgently investigated because of its pivotal role in regulating synthesis of other RNAs, including mRNA and other noncoding RNAs [11, 37]. METTL3 itself as a protein-coding gene can be modified by methylation or acetylation or other transcriptional or post-transcription regulations [38]. By evaluating the full sequence and its promoter region in UCSC website, we found highly probable acetylation and methylation sites (including H3K4Me3 and H3K27Ac, Additional file 3: Fig. S2) in the promoter region of METTL3. Moreover, CpG islands were found in the same region. All these findings demonstrated that the abnormal expression of METTL3 could be regulated by underlying mechanisms. METTL14, another methyltransferase enzyme, was found to be downregulated in CRC in both TCGA and our samples. METTL14 itself did not have methyltransferase activity, but as an RNA adaptor, it was needed for METTL3. METTL14 interacted with METTL3 to enhance the enzymatic activity of METTL3 [39]. It remained unclear how upregulated METTL3 and downregulated METTL14 together participate in tumor development. Further studies were needed to elucidate the important role of m6A modification in the oncogenesis and development of CRC.

**Fig. 7:**
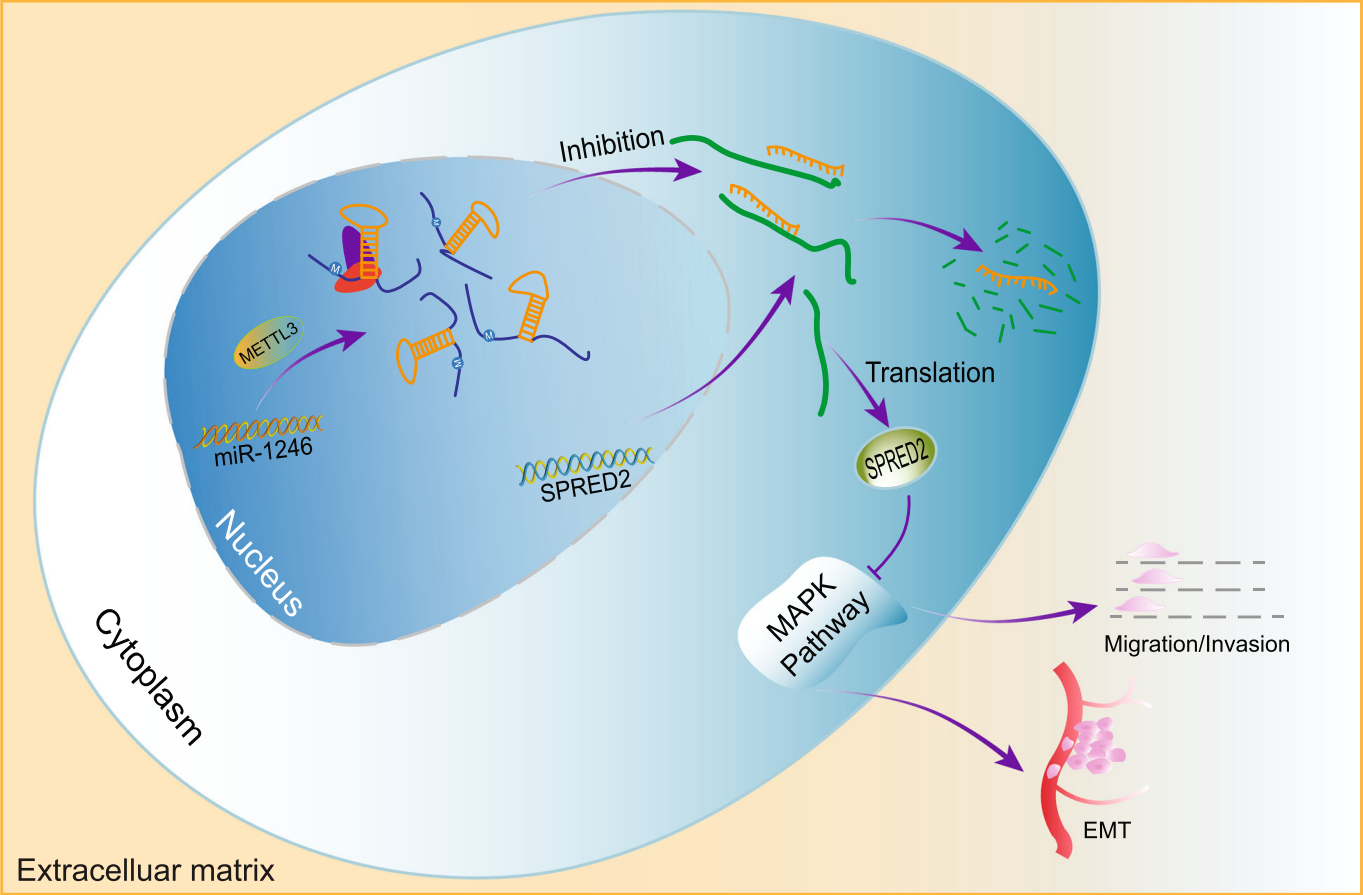
The schematic model of METTL3 in regulating CRC. METTL3 methylate pri-miR-1246 in its upstream, marking them for recognition and processing. Overexpression of METTL3 increases the level of miR-1246 by promoting the maturation of miR-1246 from pri-miR-1246. miR-1246 decreases the expression of anti-oncogene SPRED2, thus resulting in cell migration and invasion in CRC

Collectively, our findings provide a totally different mechanism of METTL3 in regulating CRC metastasis. Our study demonstrates that aberrant m6A modification in CRC was responsible for the upregulated METTL3 expression, which marks pri-miR-1246 for further processing; this increased the expression of metastasis-related miRNA-1246, resulting in downregulation of the expression of anti-oncogene SPRED2 and leading to tumor metastasis (Fig. 7).

## Conclusions

Our findings indicated that upregulated METTL3 was responsible for the abnormal m6A modification in CRC. And METTL3/miR-1246/SPRED2 axis played an important role in tumor metastasis. Importantly these results provided a new m6A modification pattern in CRC development.

## Additional files

Additional file 1: **Table S1.** Sequences of Primer for Real-time Polymerase Chain Reaction. **Table S2** Sequences of knockdown. **Table S3** Sequences of Primers for Quikchange Mutation Assay. **Table S4** Full Sequence of pri-miR-1246. (DOCX 18 kb)

Additional file 2: **Table S5.** List of Primary Antibodies Used In the study. (DOCX 17 kb)

Additional file 3: **Fig. S1** Kaplan-Meier Plotter analysis indicated that higher miR-1246 expression correlated with worse OS, using publicly available data from 160 CRC patients. **Fig. S2** Data from UCSC showed the promoter region of METTL3. CpG Islands, H3K4Me1/H3K4Me3/H3K27Ac Mark and DNase Signal were illustrated above. (DOCX 1045 kb)

Additional file 4: **Fig. S3** Knocking down the expression of METTL3 impaired the migration and invasion ability of LoVo cell in vitro. **Fig. S4** Knocking down the expression of METTL3 impaired the migration and invasion abilities of SGC-7901 cell in vitro. (DOCX 6686 kb)

## Data Availability

All data generated or analyzed during the study are included in this published article. GEO data was downloaded from the GEO datasets. (https://www.ncbi.nlm.nih.gov/gds/).

## Abbreviations

ALKBH5: alkb homolog 5
CRC: Colorectal cancer
FTO: Fat mass and obesity–associated protein
IHC: Immunohistochemical
m6A: N6-methyladenosine
MeRIP: m6A RNA immunoprecipitation
METTL3: methyltransferase-like 3
miR-1246: miRNA-1246
qRT-PCR: quantitative real-time quantitative polymerase chain reaction
RIP: RNA immunoprecipitation
siRNA: small interfering RNA
UTR: Untranslated region
