# Supplementary material for: Upregulated METTL3 promotes metastasis of colorectal Cancer via miR-1246/SPRED2/MAPK signaling pathway
Source: J Exp Clin Cancer Res. 2019 Sep 6;38:393. doi: 10.1186/s13046-019-1408-4 (PMC6729001; doi:10.1186/s13046-019-1408-4)
Supplement: Supplementary file 1 — Table S1. Sequences of Primer for Real-time Polymerase Chain Reaction. Table S2 Sequences of knockdown. Table S3 Sequences of Primers for Quikchange Mutation Assay. Table S4 Full Sequence of pri-miR-1246. (DOCX 18 kb) [file 13046_2019_1408_MOESM1_ESM.docx]

**Supplementary Table S1 Sequences of Primer for Real-time Polymerase Chain Reaction**

| **METTL3** |  |
| --- | --- |
| Forward | 5’-AGGCAGCTCATCTGTGTCCT-3’ |
| Reverse | 5’-GCTTGGCGTGTGGTCTTT-3’ |
| **SPRED2** |  |
| Forward | 5’-ATTTTTCCCTCGACACCCGAT-3’ |
| Reverse | 5’-TCCCAGGCGTAGACCAAGA-3’ |
| **β-actin** |  |
| Forward | 5’-ACTGGAACGGTGAAGGTGAC-3’ |
| Reverse | 5’-AGAGAAGTGGGGTGGCTTTT-3’ |
| **pri-miR-1246** |  |
| Forward | 5’-TGAAGTAGGACTGGGCAGAGA-3’ |
| Reverse | 5’-TTTGGGTCAGGTGTCCACTC-3’ |

**Supplementary Table S2 Sequences of knockdown**

| **siMETTL3-1** |  |
| --- | --- |
| Sense | 5’-AGGCAGCTCATCTGTGTCCT-3’ |
| Anti-sense | 5’-UAGUACGGGUAUGUUGAGCTT-3’ |
| **siMETTL3-2** |  |
| Sense | 5’-GGUUGGUGUCAAAGGAAAUTT-3’ |
| Anti-sense | 5’-AUUUCCUUUGACACCAACCTT-3’ |
| **shMETTL3** |  |
| Sense | 5’-CACCGCTGCACTTCAGACGAATTATCGAAATAATTCGTCTGAAGTG  CAGC -3’ |
| Anti-sense | 5’-AAAAGCTGCACTTCAGACGAATTATTTCGATAATTCGTCTGAAGTG  CAGC -3’ |

**Supplementary Table S3 Sequences of Primers for Quikchange Mutation Assay**

| **A-26-G** |  |
| --- | --- |
| Sense | 5’-GAGAATAATCCACATCAAGGGATCTTAGTAATGTGAACCATCAAT-3’ |
| Anti-sense  **A-57-G**  Sense  Anti-sense | 5’-ATTGATGGTTCACATTACTAAGATCCCTTGATGTGGATTATTCTC-3’  5'-CTCTTATCTCTGCCCAGCCCTACTTCATTGATGGT-3'  5'-ACCATCAATGAAGTAGGGCTGGGCAGAGATAAGAG-3' |

**Supplementary Table S4 Full Sequence of pri-miR-1246**

| >hg38_dna range=chr2:176600880-176601152  AGGGAGAATAATCCACATCAAGGGAACTTAGTAATGTGAACCATCAATGAAGTAGGACTGGGCAGAGATAAGAGACATTGCATTTGGAGGCGGTCAGATTTGTATCCTTGAATGGATTTTTGGAGCAGGAGTGGACACCTGACCCAAAGGAAATCAATCCATAGGCTAGCAATCAACCTATTTTTTGTTTTTGTAGCATTTGATCGTTATCGAGTTTTGCTGAATCCTACTTCTCTCTTTGTAAAACGGAAAAATAACAATACCTAACTCAAA |
| --- |
