# Supplementary material for: Upregulated METTL3 promotes metastasis of colorectal Cancer via miR-1246/SPRED2/MAPK signaling pathway
Source: J Exp Clin Cancer Res. 2019 Sep 6;38:393. doi: 10.1186/s13046-019-1408-4 (PMC6729001; doi:10.1186/s13046-019-1408-4)
Supplement: Supplementary file 2 — Table S5. List of Primary Antibodies Used In the study. (DOCX 17 kb) [file 13046_2019_1408_MOESM2_ESM.docx]

| Anti-Argonaute-2 antibody | Rabbit/ Polyclonal | abcam, #ab32381 | RIP: 1:100 |
| --- | --- | --- | --- |
| Anti-IgG antibody | Rabbit monoclonal | beyotime, #A7016 | RIP: 1:100 |
| Anti-m6A antibody | Mouse Monoclonal | Abcam, #ab208577 | MeRIP: 1:150 |
| Anti-SPRED2 antibody | Rabbit Polyclonal | abcam, #ab153700 | WB: 1:1000 |
| Anti-Raf antibody | Rabbit monoclonal | abcam, #ab181115 | WB: 1:1000 |
| Anti-MEK1 antibody | Rabbit monoclonal | abcam, #ab32576 | WB: 1:1000 |
| Anti-MEK1 (phospho) antibody | Rabbit monoclonal | abcam, #ab214445 | WB: 1:1000 |
| Anti-ERK1 + ERK2 antibody | Rabbit polyclonal | abcam, #ab17942 | WB: 1:1000 |
| Anti-ERK1 (phospho)  + ERK2 (phospho) antibody | Rabbit monoclonal | abcam, #ab201015 | WB: 1:1000 |
| Anti-GAPDH Antibody | Mouse Monoclonal | proteintech, #60004-1-Ig | WB: 1:5000 |
| Anti- Beta Actin Antibody | Rabbit Polyclonal | proteintech, # 20536-1-AP | WB: 1:5000 |
| Anti-METTL3 | Rabbit monoclonal | abcam, # ab195352 | WB: 1:1000 |
| Anti-METTL3 | Rabbit monoclonal | abcam, # ab195352 | IHC-P: 1:500 |

Supplementary Table S5 List of Primary Antibodies Used In the study
