# Supplementary material for: Upregulated METTL3 promotes metastasis of colorectal Cancer via miR-1246/SPRED2/MAPK signaling pathway
Source: J Exp Clin Cancer Res. 2019 Sep 6;38:393. doi: 10.1186/s13046-019-1408-4 (PMC6729001; doi:10.1186/s13046-019-1408-4)
Supplement: Supplementary file 3 — Fig. S1 Kaplan-Meier Plotter analysis indicated that higher miR-1246 expression correlated with worse OS, using publicly available data from 160 CRC patients. Fig. S2 Data from UCSC showed the promoter region of METTL3. CpG Islands, H3K4Me1/H3K4Me3/H3K27Ac Mark and DNase Signal were illustrated above. (DOCX 1045 kb) [file 13046_2019_1408_MOESM3_ESM.docx]

**Figure.S3** Kaplan-Meier Plotter analysis indicated that higher miR-1246 expression correlated with worse OS, using publicly available data from 160 CRC patients.

**Fig.S4** Data from UCSC showed the promoter region of METTL3. CpG Islands, H3K4Me1/H3K4Me3/H3K27Ac Mark and DNase Signal were illustrated above.
