# Supplementary material for: Upregulated METTL3 promotes metastasis of colorectal Cancer via miR-1246/SPRED2/MAPK signaling pathway
Source: J Exp Clin Cancer Res. 2019 Sep 6;38:393. doi: 10.1186/s13046-019-1408-4 (PMC6729001; doi:10.1186/s13046-019-1408-4)
Supplement: Supplementary file 4 — Fig. S3 Knocking down the expression of METTL3 impaired the migration and invasion ability of LoVo cell in vitro. Fig. S4 Knocking down the expression of METTL3 impaired the migration and invasion abilities of SGC-7901 cell in vitro. (DOCX 6686 kb) [file 13046_2019_1408_MOESM4_ESM.docx]

Fig. S3

**Figure S3** **Knocking down the expression of METTL3 impaired the migration and invasion ability of LoVo cell in vitro.** (A) The knockdown efficiency was determined by qRT-PCR (upper panel) and western blot (lower panel). (B) Effect of knocking down the expression of METTL3 on transwell assay; Representative graphs are shown. Original magniﬁcation 200X; scale bar: 50μm. (C) Effect of knocking down METTL3 level on scratching healing assay; The scratch was measured 48 hours later; Representative graphs are shown. Original magnification, 40X; scale bar: 100μm. Data are presented as means ± standard deviation (**P < 0.01, ***P<0.001).

Fig. S4

**Figure S4** **Knocking down the expression of METTL3 impaired the migration and invasion abilities of SGC-7901 cell in vitro.** (A) The knockdown efficiency was determined by qRT-PCR (left panel) and western blot (right panel). (B) Cell counting kit-8 assay. (C) Effect of knocking down the expression of METTL3 on transwell assay; Representative graphs are shown. Original magniﬁcation 200X; scale bar: 50μm. (D) Effect of knocking down METTL3 level on scratching healing assay; The scratch was measured 48 hours later; Representative graphs are shown. Original magnification, 40X; scale bar: 100μm. Data are presented as means ± standard deviation (**P < 0.01, ***P<0.001).
